# Popliteal deep vein thrombosis in a pediatric patient with S*treptococcus viridans* osteomyelitis: a case report

**DOI:** 10.1093/jscr/rjae758

**Published:** 2024-12-05

**Authors:** Joshua Hansen, Micah Ngatuvai, Alexys Bermudez, Joshua Salisbury, Kyle Klahs, Gilbert Handel, Amr Abdelgawad, Ahmed M Thabet

**Affiliations:** Texas Tech University of the Health Sciences, El Paso, Department of Orthopaedic Surgery and Rehabilitation, 4801 Alberta Ave, El Paso, TX 79905,United States; Texas Tech University of the Health Sciences, El Paso, Department of Orthopaedic Surgery and Rehabilitation, 4801 Alberta Ave, El Paso, TX 79905,United States; Tripler Army Medical Center, Department of Orthopaedic Surgery, 1 Jarrett White Rd, TRIPLER AMC, HI 96859,United States; Texas Tech University of the Health Sciences, El Paso, Department of Orthopaedic Surgery and Rehabilitation, 4801 Alberta Ave, El Paso, TX 79905,United States; Texas Tech University of the Health Sciences, El Paso, Department of Orthopaedic Surgery and Rehabilitation, 4801 Alberta Ave, El Paso, TX 79905,United States; Texas Tech University of the Health Sciences El Paso, Paul L Foster School of Medicine, 5001 El Paso Dr, El Paso, TX 79905, United States; Texas Tech University of the Health Sciences, El Paso, Department of Orthopaedic Surgery and Rehabilitation, 4801 Alberta Ave, El Paso, TX 79905,United States; Texas Tech University of the Health Sciences, El Paso, Department of Orthopaedic Surgery and Rehabilitation, 4801 Alberta Ave, El Paso, TX 79905,United States

**Keywords:** infection, deep vein thrombosis, osteomyelitis, pediatrics

## Abstract

An 11-year-old male presented with severe pain in the left knee after a minor twisting injury. Magnetic resonance imaging (MRI) revealed a large fluid collection posterior to the distal femur. CT and intraoperative findings revealed left popliteal vein thrombosis. Irrigation and debridement were performed, and intraoperative cultures confirmed osteomyelitis due to pan-sensitive *Streptococcus viridans (S. viridans)*. Treatment included antibiotic and anticoagulation therapy. At 6 months, the patient was asymptomatic and cleared for full activity. This case demonstrates that deep vein thrombosis (DVT) can occur with *S. viridans* osteomyelitis, a previously undescribed association. Clinicians should maintain suspicion for DVT with any causative organism in osteomyelitis.

## Introduction

Acute osteomyelitis occurs in ~8 of 100 000 children per year in Western countries. The etiology includes traumatic wounds, contiguous spread from adjacent tissues, or through hematogenous seeding [1]. The majority of infections in otherwise healthy children occur secondary to bacteremia and hematogenous seeding into the metaphysis of long bones, with the femur and tibia being the most affected regions [2–4].

The most common causative agent of osteomyelitis is *Staphylococcus aureus (S. aureus)*, with up to 80% of cases resulting in positive cultures [2, 3, 5]. Deep venous thrombosis (DVT) is a reported complication of pediatric osteomyelitis and is associated with *S. aureus* infections (both MRSA and MSSA), and has been increasing in incidence within the last decade [6]. The high occurrence of DVT with *S. aureus* infection likely occurs secondary to the variable presence of the Panton Valentine Leukocidin (*pvl*) gene, which produces an exotoxin that has been implicated in platelet aggregation, facilitating thrombosis [4, 7].

This case report describes an 11-year-old male patient with homogenously spread distal femoral osteomyelitis with positive cultures for *Streptococcus viridans* and superimposed ipsilateral popliteal vein DVT. This study aimed to provide a case demonstrating the atypical presentation and course of pediatric distal femur osteomyelitis with DVT resulting from a previously undescribed bacterium.

## Patient course

**Figure 1:**
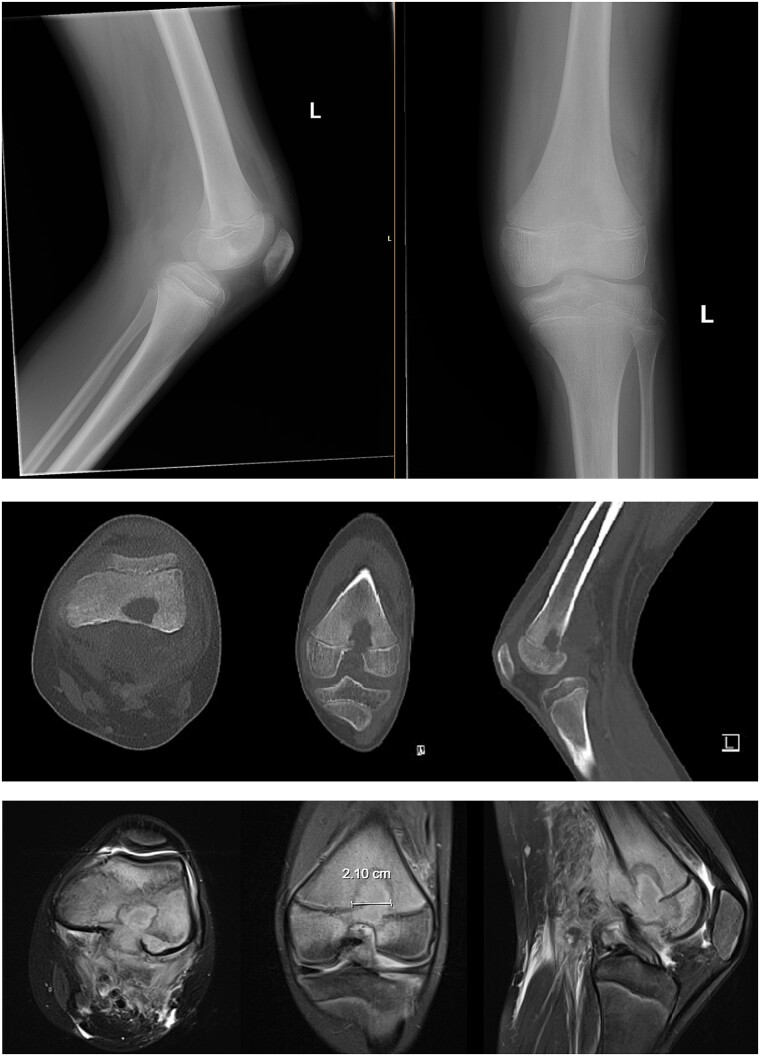
Preoperative radiography, CT, and MRI. The radiographs were read as normal by the radiology, while the CT and MRI were read osteomyelitis with Brodie’s abscess involving the distal femoral epimetaphysis with destruction of the posterior cortex and associated 7 cm popliteal fossa abscess, along with a suspected nonocclusive septic thrombophlebitis of the popliteal vein mentioned in the report on the CT.

An 11-year-old healthy male presented with posterior left knee pain, which began 4 days after twisting his knee at a trampoline park. The pain progressed to a point where he was unable to bear weight on his left leg, and the knee became locked in flexion due to muscle spasms and pain. After three visits to outside facilities over 2 weeks, the patient finally received an orthopedic consultation. While radiographs were unremarkable, magnetic resonance imaging (MRI) revealed a large fluid collection posterior to the distal femur, suggestive of osteomyelitis, and Brodie’s abscess and associated local destruction of the posterior femoral cortex (Fig. 1a and c). The patient was then transferred to our institution for definitive management. Upon presentation, the patient was febrile (39°C) and nauseous with one episode of emesis. Physical examination revealed a tender and warmer left knee without open wounds. The patient denied a knowledge of current dental carries or a history of recent dental procedures and denied recent history of a streptococcal infection. Laboratory tests showed leukocytosis of 14 600 μl of white blood cells (WBC), elevated C-reactive protein (CRP) of 18 μg/ml (normal <0.3 μg/ml), and an elevated erythrocyte sedimentation rate (ESR) of 18 mm/h (normal <15 mm/h). A CT scan at our institution recapitulated findings suggestive of osteomyelitis, cortical destruction, and a noted thrombophlebitis of the popliteal vein (Fig. 1b).

The patient was admitted to the children’s hospital and was started on broad-spectrum intravenous antibiotics. A left knee synovial aspiration was performed, which yielded normal results. A venous ultrasound was performed but was deemed technically unsatisfactory for interpretation. On hospital Day 1, the patient underwent left distal femur irrigation and debridement (I&D) with intraoperative cultures obtained. During the surgical dissection, the popliteal vein was found to be non-compressible, consistent with DVT, prompting the initiation of anticoagulation therapy (rivaroxaban, 15 mg daily) alongside appropriate antibiotic therapy.

**Figure 2:**
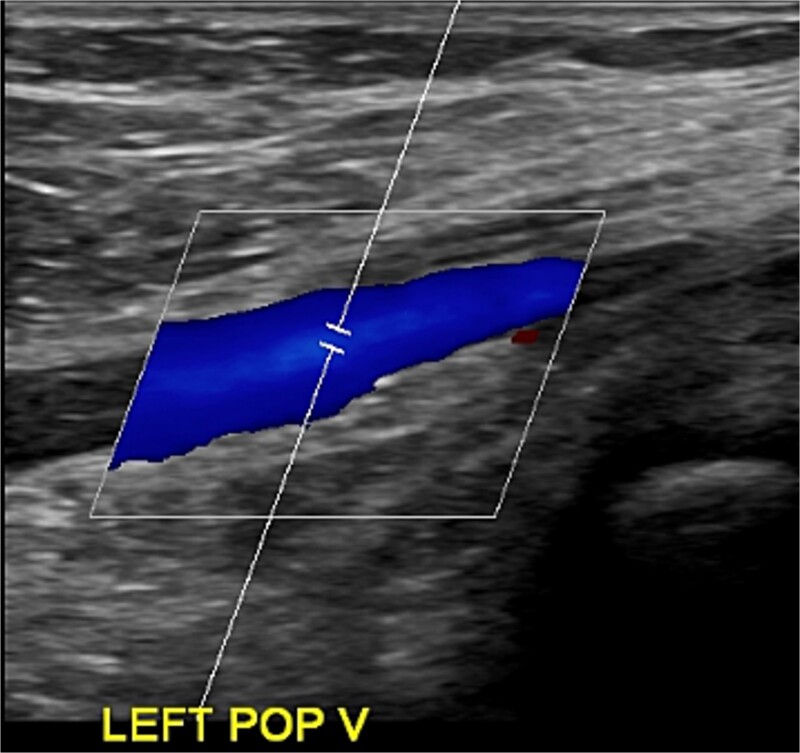
Ultrasonography obtained in January of 2023 demonstrating resolution of DVT.

**Figure 3:**
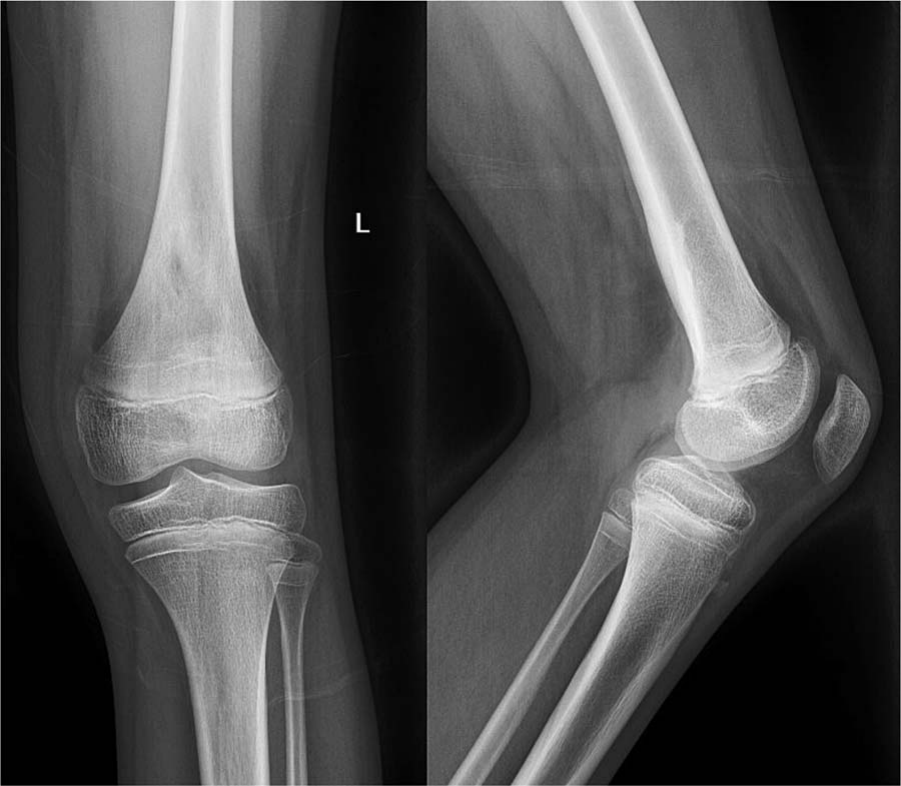
Radiography obtained 1-year postoperatively, demonstrating no acute osseous changes.

**Figure 4:**
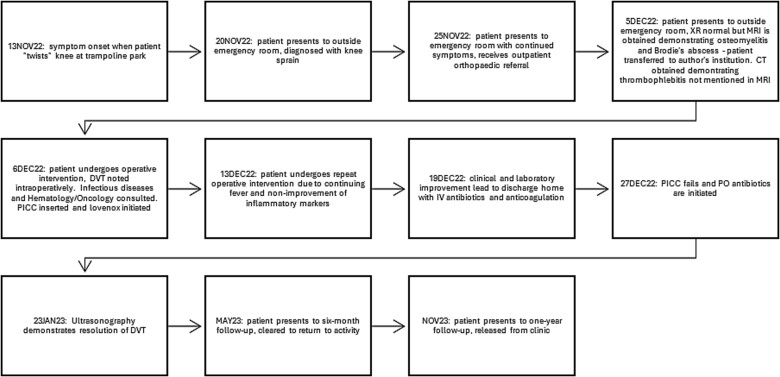
Treatment timeline, beginning with the onset of symptoms and the ending with the patient’s follow-up at 1 year.

Despite initial improvement, the patient’s CRP stabilized at 5.5 μg/ml 6 days post-operatively. Consequently, a repeat I&D with antibiotic bead placement was performed on postoperative Day 7. The patient’s condition improved, with undetectable CRP levels at discharge 14 days after initial hospitalization. Four weeks post-discharge, a repeat venous ultrasound of the affect extremity was performed, demonstrating resolution of the DVT (Fig. 2). In total, the patient received 18 weeks of outpatient oral antibiotic therapy and 9 weeks of anticoagulation therapy. Six months post-hospitalization, the patient was asymptomatic and cleared to return to full activity. After 1-year post-discharge, final radiographs demonstrated no osseous abnormalities, the patient remained asymptomatic, and was released from clinic (Figs 3 and 4).

## Discussion

Deep venous thrombosis in the pediatric population is exceedingly rare, with an estimated incidence ranging from 0.07 to as high as 21.9 in 10 000 [8, 9]. This case is especially unique as no association between DVT and *S. viridans* has been previously described. The patient presented to multiple emergency rooms with ill-defined pain and was eventually appropriately diagnosed and treated with surgical and medical interventions, which allowed for successful eradication of the infection and DVT.

Across multiple studies, ~10% of children with various musculoskeletal infections develop DVT, with osteomyelitis being the most frequently associated [4]. Most DVTs are located in the lower extremities adjacent to the infected bone [6, 7]. The association of pediatric DVT and osteomyelitis infection with *S. aureus*, specifically MRSA, has been well documented, with some studies reporting positive cultures in all patients with acute hematogenous osteomyelitis and associated DVT [9]. *Streptococcus viridans*, the causative organism in this patient, is a less common cause of osteomyelitis, and no published literature has reported a connection between *S. viridans* and DVT. The prothrombotic state of this patient may have simply occurred secondary to inflammatory mediators that were a result of the immune system’s natural response to bacterial surface proteins, which promote platelet aggregation and endothelial dysfunction [6].

Common symptoms of osteomyelitis sometimes overlap with the presenting symptoms of DVT. With the increased frequency of DVT associated with osteomyelitis, it is important for clinicians to have a low threshold for initiating an appropriate diagnostic workup for DVT using Doppler sonography. One study found that patients with osteomyelitis who developed DVT had higher inflammatory markers (ESR and CRP) upon admission than those who did not develop DVT [7]. This aligns with our patient’s presentation, who had elevated inflammatory markers (CRP 18 μg/ml, ESR 18 mm/h) at the time of admission.

Previous studies have reported the use of low molecular weight heparin and warfarin for the treatment of DVT in this patient population, with 28% of patients showing signs of complete resolution within 1 week of treatment initiation [6]. Our patient was treated with Lovenox 44 mg every 12 hours for 1 week and was then transitioned to Xarelto 15 mg once daily, which was continued for 8 weeks. The patient was seen for follow-up 1 month after discharge and had full resolution of symptoms after 6 months, as evidenced by follow-up MRI and serial ultrasound.

## Conflict of interest statement

None declared.

## Funding

None declared.

## Statement of informed consent

The patient and his guardian were informed that the data concerning the case would be submitted for publication. All patients agreed and consented to release the data for publication.

## References

[1] Riise ØR , Kirkhus E, Handeland KS, et al. Childhood osteomyelitis-incidence and differentiation from other acute onset musculoskeletal features in a population-based study. BMC Pediatr 2008;8:45. 10.1186/1471-2431-8-45. 18937840 PMC2588573

[2] Peltola H , Pääkkönen M. Acute osteomyelitis in children. N Engl J Med 2014;370:352–60. 10.1056/NEJMra1213956. 24450893

[3] Iliadis AD , Ramachandran M. Paediatric bone and joint infection. EFORT Open Rev 2017;2:7–12. 10.1302/2058-5241.2.160027. 28607765 PMC5444236

[4] Mantadakis E , Plessa E, Vouloumanou EK, et al. Deep venous thrombosis in children with musculoskeletal infections: the clinical evidence. Int J Infect Dis IJID Off Publ Int Soc Infect Dis 2012;16:e236–43. 10.1016/j.ijid.2011.12.012. 22361432

[5] Thakolkaran N , Shetty AK. Acute Hematogenous osteomyelitis in children. Ochsner J 2019;19:116–22. 10.31486/toj.18.0138. 31258423 PMC6584206

[6] Citla Sridhar D , Maher OM, Rodriguez NI. Pediatric deep venous thrombosis associated with staphylococcal infections: single institutional experience. J Pediatr Hematol Oncol 2018;40:e73–6. 10.1097/MPH.0000000000001040. 29200147

[7] Bouchoucha S , Benghachame F, Trifa M, et al. Deep venous thrombosis associated with acute hematogenous osteomyelitis in children. Orthop Traumatol Surg Res OTSR 2010;96:890–3. 10.1016/j.otsr.2010.05.006. 20833120

[8] Schaub RL , Rodkey ML. Deep vein thrombosis and septic pulmonary emboli with MRSA osteomyelitis in a pediatric patient. Pediatr Emerg Care 2012;28:911–2. 10.1097/PEC.0b013e318267ea4e. 22940890

[9] Ligon JA , Journeycake JM, Josephs SC, et al. Differentiation of deep venous thrombosis among children with or without osteomyelitis. J Pediatr Orthop 2018;38:e597–603. 10.1097/BPO.0000000000001240. 30080773

